# Factors influencing the development of primary care data collection projects from electronic health records: a systematic review of the literature

**DOI:** 10.1186/s12911-017-0538-x

**Published:** 2017-09-25

**Authors:** Marie-Line Gentil, Marc Cuggia, Laure Fiquet, Camille Hagenbourger, Thomas Le Berre, Agnès Banâtre, Eric Renault, Guillaume Bouzille, Anthony Chapron

**Affiliations:** 1 Department of General Practice, University of Rennes 1, F-35000 Rennes, France; 2 CIC (Clinical investigation center) INSERM 1414, F-35000 Rennes, France; 3 INSERM, U1099, F-35000 Rennes, France; 4 University of Rennes 1, LTSI (Laboratory for signal and image processing), F-35000 Rennes, France; 5 CHU Rennes, CIC Inserm 1414, F-35000 Rennes, France; 6 CHU Rennes, Centre de Données Cliniques, F-35000 Rennes, France

**Keywords:** Primary care, Data mining, Data collection, Secondary use, Electronic health records, Governance, Stakeholders

## Abstract

**Background:**

Primary care data gathered from Electronic Health Records are of the utmost interest considering the essential role of general practitioners (GPs) as coordinators of patient care. These data represent the synthesis of the patient history and also give a comprehensive picture of the population health status. Nevertheless, discrepancies between countries exist concerning routine data collection projects. Therefore, we wanted to identify elements that influence the development and durability of such projects.

**Methods:**

A systematic review was conducted using the PubMed database to identify worldwide current primary care data collection projects. The gray literature was also searched via official project websites and their contact person was emailed to obtain information on the project managers. Data were retrieved from the included studies using a standardized form, screening four aspects: projects features, technological infrastructure, GPs’ roles, data collection network organization.

**Results:**

The literature search allowed identifying 36 routine data collection networks, mostly in English-speaking countries: CPRD and THIN in the United Kingdom, the Veterans Health Administration project in the United States, EMRALD and CPCSSN in Canada. These projects had in common the use of technical facilities that range from extraction tools to comprehensive computing platforms. Moreover, GPs initiated the extraction process and benefited from incentives for their participation. Finally, analysis of the literature data highlighted that governmental services, academic institutions, including departments of general practice, and software companies, are pivotal for the promotion and durability of primary care data collection projects.

**Conclusion:**

Solid technical facilities and strong academic and governmental support are required for promoting and supporting long-term and wide-range primary care data collection projects.

## Background

The secondary use of Electronic Health Record (EHR) data, for instance for epidemiological research, pharmacovigilance or health policy making, is progressively increasing [1]. Moreover, due to the chronic nature of many diseases, a global understanding of the patient’s history is crucial for quality healthcare. In this respect, a paradigm shift occurred with the development of Big Data analysis following EHR digitization that facilitates data processing. Indeed, data mining brings large amount of information with higher granularity (i.e., higher level of detail).

In France, several data retrieval projects [2–4] are currently focused on the collection and mining of hospital administrative data (for instance, the Program of Medicalization of the Information Systems) and of clinical data from hospital EHRs. Data retrieved from hospital sources are promising, but they do not take into account the entire care pathway of each single patient or of the whole population. From this point of view, primary care records are particularly interesting [5]. Indeed, as general practitioners (GPs) are often the coordinators for their patients’ healthcare trajectory, primary care records should contain the entire medical history of each patient [6]. Moreover, most people have access to primary care. Thanks to information technologies (IT), the volume of data captured by EHRs, paired with the growing capacity for data linkage and exchange, creates opportunities for measuring outcomes and, consequently, for improving patient and population health. In France, one such initiative was the “Observatoire de la Médecine Générale” (a nationwide survey of GPs’ practice) that ended in 2009 due to lack of funding. Currently, the French Institute for Research and Documentation on Health Economics (IRDES) exploits the primary care data obtained by other companies, such as IMS Health©, a private-sector firm [7], via partnership agreements. Nevertheless, the lack of information on how these data were collected raises methodological concerns [7]. Cegedim©, another private-sector company, works on data extracted from French primary care EHRs [8]. Few local initiatives also have been implemented [9], but we could not find a transparent French national infrastructure that collects data directly from primary care practices. Conversely, in other countries, the possible contribution to medical science and health policy decision-making of routinely collected primary care data is now assessed, for instance with the Clinical Practice Research Datalink (CPRD) in the United Kingdom (UK) [10, 11].

Considering the discrepancies between countries on routine data collection from primary care EHRs, we wanted to identify factors that might facilitate the development and durability of routine primary care data collection. To this aim, we reviewed primary care data collection projects worldwide by taking into account their technical features, the GPs’ contribution and the network managers.

## Methods

A systematic review of the literature was performed from December 2015 to November 2016, based on the Preferred Reporting Items for Systematic Reviews and Meta-Analyses (PRISMA [12]) criteria.

The checklist points for Assessing the Methodological Quality of Systematic Reviews (AMSTAR [13]) were completed when criteria were relevant, that is to say points 1 to 5, 10 and 11. We referred to it at the beginning to define our work protocol (point 1), duplicate study selection and data extraction (point 2), perform a comprehensive literature search (point 3 and 5). Then it guided us through our analysis of included papers: status of publication (point 4), scientific quality, publication bias (point 10) and conflict of interest (point 11).

### Information sources

First, an automated literature search of the PubMed database was performed with the assistance of a university librarian, with expertise in systematic reviews.

**Fig. 1:**
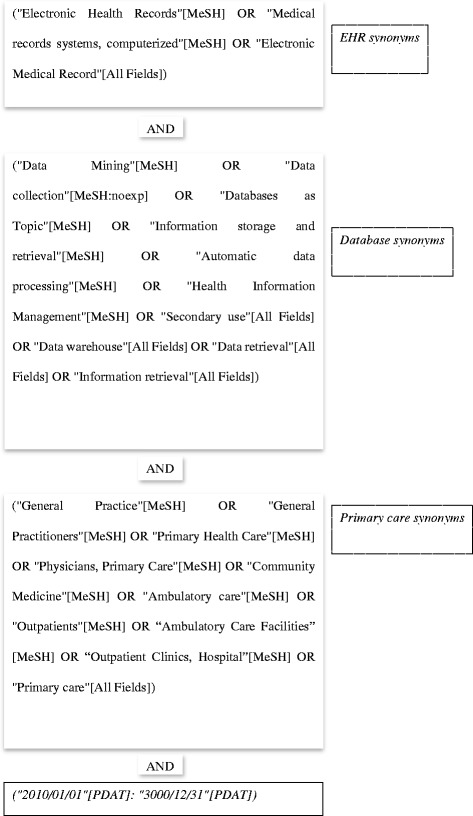
PubMed query

To identify data collection projects based on primary care EHRs, worldwide, our query was divided in three parts: i) collection of EHR synonyms; ii) retrieval of records about automatic data processing; and iii) identification of primary care data collection projects, using several MeSH term synonyms. To expand our search, MeSH terms and also free text words were used. Articles published from 2010 onwards were selected. No other filter was applied. The following query (Fig. 1) was submitted to the PubMed search engine. The last search was performed in November 2016.

Secondary to our PubMed search, we identified 36 routine data collection projects. Our screening of the gray literature consists of the screening of each project website. First, we tried to find the official websites from our literature review. Then, when the website was not quoted in the references of the paper, we used the Google search engine. The name of the database was associated with the keywords “database” or “primary care database” enclosed in quotation marks.

Moreover, to identify the project managers, the project contact person quoted in the website was contacted by email, when such information was present in the article/website.

### Study selection

#### Inclusion criteria

Articles were retained if they referred exclusively to EHRs (as opposed to paper-based health records), focused on automatic data processing (rather than on manual data analysis) and the EHRs were retrieved from primary care databases. Secondary or tertiary care data were not considered. Within primary care data, raw patient medical records were included, while registries, which contain already processed data, were excluded. Databases containing primary and secondary care data were included only if the study concerned the primary care population. An article was considered to be a routine primary care data collection project when it analyzed EHR data from different GPs.

#### Exclusion criteria

Articles published before 2010 were excluded due to the introduction of new MeSH terms, for instance “Electronic Health Record”, in 2010. The 2010 cutoff was indeed primarily used due to the introduction of more relevant MeSH headings in 2010 so as to define active databases. It allowed us to retrieve active routine data collection projects, because they published papers recently and our PubMed query was more accurate thanks to the MeSH term indexing.

Moreover, we excluded:

- Articles not written in French nor English languages
- Articles on projects not meeting the inclusion criteria
- Articles that could not be retrieved as full text, due to an absence of subscription to the review
- Articles in which the original database was not precisely identified.

### Data collection

The PubMed search query was launched independently by two of the authors who then read the abstracts to select relevant publications on the basis of the inclusion and exclusion criteria. They then independently read the full text of the retained articles to confirm that the inclusion and exclusion criteria were met. Disagreements were solved by consensus.

**Fig. 2:**
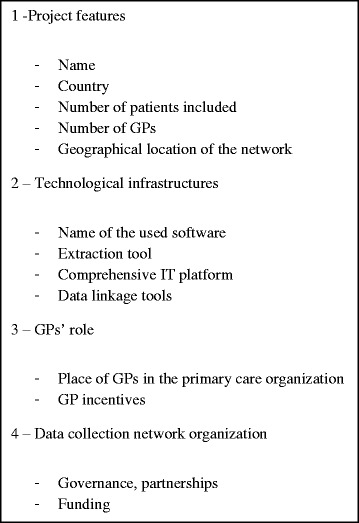
Form used to analyze the selected primary care data collection projects and relevant websites

This allowed us to identify the major primary care data collection projects and to compare their prevalence worldwide. Each PubMed article was screened to retrieve information on the data collection project and its stakeholders with the objective to compare projects. Data were retrieved from the included studies using a standardized form (Fig. 2). This form was based on that of a Canadian qualitative study on the use of primary healthcare EHRs for research [14], and was revised by the authors during a working discussion. Indeed, this paper allowed us to extrapolate three parts of our form: technological infrastructure, GPs’ roles, data collection network stakeholders. The form was pre-tested on 5% of the selected papers and was not modified after this test. Two authors read separately seven articles and filled in the form. Then, they compared their results. There were no disagreements due to the objective nature of the data extracted. Consequently, the form was left unmodified.

Each official project website also was screened using the same form. Moreover, each contact person received one single e-mail message to gather information about the data collection project partners (identification of the involved parties).

Three groups of stakeholders were defined from the various partners retrieved in papers and websites: governmental services, academic institutions, software companies, so as to represent each identified extracted partnership. The definition of stakeholders groups was ascertained by [12], for instance we can quote “Within this study, we define stakeholders as those individuals holding an interest in the topic of EMRs in PHC; these individuals included clinicians/healthcare practitioners, decision-makers (those who make policy and health planning decisions), researchers and EMR vendors.”

## Results

### Selection of articles on primary care data collection projects

**Fig. 3:**
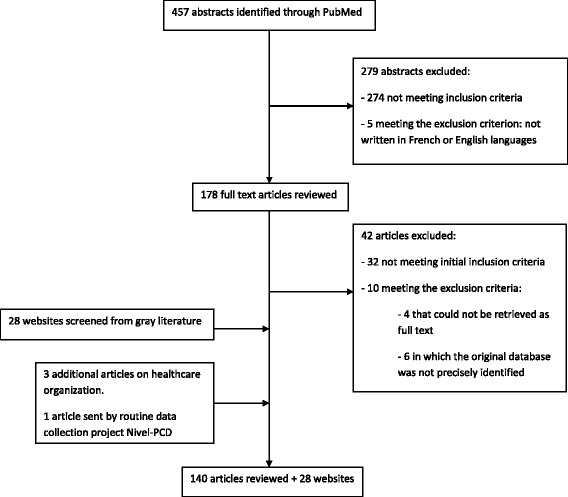
Article selection flow chart

The PubMed search yielded 457 article abstracts among which 279 were not retained based on the inclusion/exclusion criteria (Fig. 3). The remaining 178 articles dealt with primary care routine data collection projects. Another 42 articles were excluded based on the inclusion and exclusion criteria. Three articles relevant for understanding the healthcare organization and one paper sent by Nivel-Primary Care Database (Nivel-PCD) were added for the final analysis. Each contact person from the 28 websites identified by screening the gray literature was emailed to ask information about the data collection project stakeholders. The Information System for the Development of Primary Care Research (SIDIAP) and Canning Division of General Practice contacts were not operational. We received ten answers and no follow-up email message was sent.

### Comparison of the selected routine primary care data collection projects

#### List of routine primary care data collection projects

**Fig. 4:**
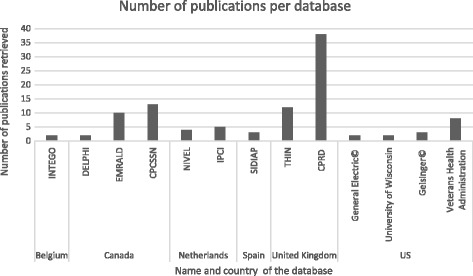
Number of publications per database. Projects with at least two publications retrieved by our query

Review of the retained articles allowed the identification of 36 projects on collection of data from primary care EHRs (Table 1). They were mainly from English-speaking countries (USA, UK, Canada, Australia) as shown by Fig. 4, but also from various European countries. The TRANSFoRm project involved different European countries.

**Table 1:** List of the routine primary care data collection projects

Project name or network associated with the database	Country of origin	PubMed occurrence (ref)	Official website (ref)
Geographical and Resource Analysis in Primary Health Care (GRAPHC)	Australia	1 [48]	[49]
Canning Division of General Practice in Western Australia project	Australia	1 [50]	No website
Melbourne East General Practice Network in Victoria project (MEGPN)	Australia	1 [17]	[27]
Local initiative: three general practices	Australia	1 [51]	No website
Intego Project	Belgium	2 [52, 53]	[54]
National Scale Routine Data Collection Network (NIHDI)	Belgium	1 [23]	No website
Canadian Primary Care Sentinel Surveillance Network (CPCSSN)	Canada	13 [36, 55–66]	[24]
Deliver Primary Healthcare Information (DELPHI)	Canada	2 [25, 67]	[24]
Electronic Medical Record Administrative Data Linked Database (EMRALD)	Canada	10 [34, 35, 37, 62, 68–73]	[74]
One community health center – local experiment	Canada	1 [18]	No website
Longitudinal Patient Data Network - Cegedim©	France	1 [8]	Commercial website
Cegedim© Strategic Data - Longitudinal Patient Database (CSD)	Italy	3 [38, 75, 76]	[77]
TransHIS - TRANSFoRm Project - Translational Research and Patient Safety in Europe	Malta	1 [78]	[79]
TransHIS – TRANSFoRm Project - Translational Research and Patient Safety in Europe	Netherlands	1 [78]	[79]
Integrated Primary Care Information Project (IPCI)	Netherlands	5 [38, 76, 80–82]	[29]
Nivel: Primary Care Database (NIVEL-PCD) and LINH Database (Netherlands Information Network of General Practice)	Netherlands	4 [83–86]	[41]
Julius Clinical Primary Care Research Network (JPCRN)	Netherlands	1 [87]	[39]
Registration Network Family Practices (RNH)	Netherlands	1 [88]	[89]
SIDIAP, Information System for the Development of Primary Care Research	Spain	3 [90–92]	[92]
Electronic Clinical Record in Primary Care from Madrid	Spain	1 [93]	No website
BIFAP, Database for Pharmacoepidemiology	Spain	1 [94]	[95] – (website excluded because written in Spanish)
Swedestar, EHR system for primary care	Sweden	1 [96]	No website
Family Medicine ICPC-Research using Electronic Medical Records (FIRE project)	Switzerland	1 [97]	[98] –(website excluded because written in German)
Clinical Practice Research Datalink (CPRD) or General Practice Research Database (GPRD)	United Kingdom	38 [10, 40, 99–134]	[15]
The Health Improvement Network (THIN)	United Kingdom	12 [11, 135–145]	[16]
Q Research	United Kingdom	1 [11]	[42]
Local initiatives or uncompleted project, i.e. General Practice Extraction Service	United Kingdom	6 [146–151]	No website
Veterans Administration – Corporate Data Warehouse	United States- *Several states*	8 [152–159]	[28]
Geisinger© Health System Project	United States- *Several states*	3 [160–162]	[30]
General Electric© Centricity Electronic Medical Records Research Database	United States- *Several states*	2 [163, 164]	Commercial website
OCHIN, Inc. Oregon Community Health Information Network	United States - *Several states*	1 [22]	[165]
Baylor Health Care System (BHCS) and Christiana Care Health System (CCHS)	United States	1 [166]	[167]
University of California Davis Health System Electronic Medical Record System	United States – *California*	1 [168]	[169]
CHCI, Community Health Center, Inc.	United States – *Connecticut*	1 [170]	[171]
Massachusetts General Physicians Organization, MGPO	United States –*Massachusetts*	1 [19]	[172]
4-site federally qualified CHC, Open Door Family Medical Centers (Open Door), located in New York	United States – *New York*	1 [173]	[174]
New York City Primary Care Information Project	United States –*New York*	1 [21]	[175]
Institute for Family Health (IFH)	United States – *New York area*	1 [20]	[176]
Isolated initiative: Dallas (Texas)	United States – *Texas*	1 [177]	No website
University of Wisconsin Department of Family Medicine Clinical Data Warehouse	United States – *Wisconsin*	2 [178, 179]	Unavailable website
University of Wisconsin Electronic Health Record - Public Health Information Exchange (UW eHealth - PHINEX)		1 [33]

**Fig. 5:**
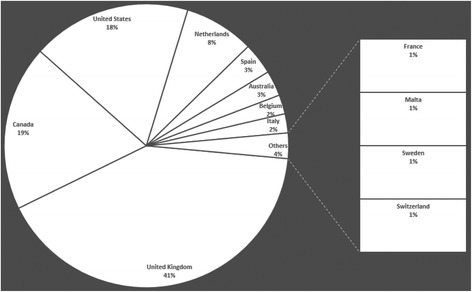
Chart representing the percentage of publications per country

By analyzing the percentage of retrieved publications (i.e., the number of papers per country extracted in our query divided by the total number of papers), countries could be classified in four groups (Fig. 5).The first group included the UK that had the highest percentage of publications compared with all the other countries (40% of all retrieved publications). Canada (19%) and the United States of America (USA) (18%) formed the second group. The third group included few European countries (Netherlands, Spain, Belgium and Italy) and Australia. These countries had successful primary data collection projects, although they were not published on as much as the other groups (2 to 8.5% of all retrieved publications). The last group comprised four European countries (France, Malta, Sweden and Switzerland) that had very few primary data collection networks.

Analysis of the publications linked to a specific primary data collection project within a country showed that the UK hosted several projects, among which CPRD (38 papers retrieved) and The Health Improvement Network (THIN) (12 papers retrieved) were the most used (Table 1). Moreover, according to their official websites, research using CPRD and THIN data has led to more than 1500 and 500 publications [15, 16], respectively. In Canada, the most quoted were the Canadian Primary Care Sentinel Surveillance Network (CPCSSN), which includes the Deliver Primary Healthcare Information **(**DELPHI) program, and the Electronic Medical Record Administrative Data Linked Database (EMRALD) project. In the USA, the Veterans Health Administration was the main provider of publications. In continental Europe, the Integrated Primary Care Information Project (IPCI) and Nivel-PCD in the Netherlands were associated with a significantly higher number of publications compared with other European projects. The Spanish SIDIAP also was quoted several times. In Italy, all projects were managed by the same private-sector company: Cegedim Strategic Data**©**. Australia had several local and apparently independent initiatives. Belgium had two databases: Intego and the National Scale Routine Data Collection Network **(**NHIDI).

This analysis allowed identifying major routine primary care data collection projects: CPRD, THIN, the Veterans Affairs Corporate Data Warehouse, CPCSSN and EMRALD.

#### Patients and healthcare professionals’ coverage

**Fig. 6:**
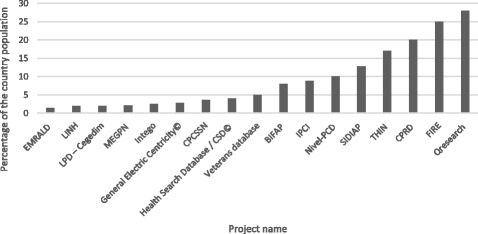
Representativeness of population in projects. Projects collecting data on more than one percent of the country population

The number of patients in the database and the number of involved GPs or practices (two parameters that are representative of the data collection project scale) are summarized in Table 2. Primary care data collection projects in the UK, Netherlands, Spain and Switzerland included the highest numbers of patients as represented in Fig. 6.

**Table 2:** Demographics of the primary care data collection projects

Country	Project name	Number of patients (% population)^a^	Number of GPs (other healthcare measurement)	Sources
UK	CPRD	13 million (20%)	600 GPs	[15]
	THIN	11.1 million (17%)	562 GPs	[16]
	Qresearch	18 million (28%)	1000 GPs	[42]
USA	Veterans database	17 million (5%)	GPs unknown (53,000 healthcare professionals)	[154]
	Geisinger© Health System project	400,000 (0.1%)	GPs unknown (41 community practice clinics)	
	General Electric Centricity©	8.9 million (2.8%)	7259 GPs	[163, 164]
	OCHIN, Inc	1 million patients annually (0.3%)	GPs unknown (200 clinic sites)	[22]
	Baylor Health Care System (BHCS) and Christiana Care Health System (CCHS)	680,000 patients (0.2%)	GPs unknown (33 primary care practices + 35 office practices)	[166]
	University of California Davis Health System’s electronic medical record system	unknown	GPs unknown (13 clinics in this analysis)	[168]
	CHCI – Connecticut	130,000 (0.04%)	GPs unknown (13 primary health centers)	[170]
	MGPO	87,568 patients in this study (0.02%)	148 GPs in this study	[19]
	OpenDoor	40,000 patients annually (0.01%)	GPs unknown (4-site federally qualified community health center in New York)	[173]
	New York City Primary Care Information Project	unknown	GPs unknown (82 practices)	[21]
	IFH	90,000 patients (0.02%)	GPs unknown (17 community health centers)	[20]
	Department of Family Medicine – Wisconsin	110,000 for these studies (0.03%)	GPs unknown (28 primary care clinics)	[178, 179]
Canada	CPCSSN	1.28 million (3.6%)	1031 GPs	[24, 56]
	EMRALD	0.5 million (1.4%)	350 GPs	[74]
	DELPHI	30,000 (0.08%)	30 GPs	[24]
Netherlands	IPCI	1.5 million (8.8%)	600 GPs	[29, 38]
	Nivel-PCD LINH	1.6 million (10%) 350,000 (2%)	GPs unknown (500 practices) 85–150 GPs	[41, 83, 85]
	Julius	156,176 (0.9%)	60 GPs	[41, 87]
	RNH	51,700 (0.3%)	65 GPs (22 practices)	[89]
Spain	SIDIAP	6 million (12.8%)	274 GPs	[90]
	ECRPC	unknown	GPs unknown	
	BIFAP	3.2–4.8 million (6.8–10%)	1183 GPs	[94, 95]
Australia	GRAPHC	32,000 (0.1%)	GPs unknown (1 GP practice)	[48]
	Canning Division of General Practice	unknown	GPs unknown (8 GP sites)	[50]
	MEGPN	505,600 (2.1%)	578 GPs (141 practices)	[17]
Belgium	Intego	285,000 (2.5%)	GPs unknown (95 GP practices)	[52]
	NIHDI	29,000 (0.25%)	GPs unknown (5000 practices)	[23]
France	LPD – Cegedim	1.5 million (2%)	1200 GPs (+ 750 various medical specialists)	[8]
Italy	Health Search Database / CSD©	2.5 million (4.1%)	900 GPs	[77]
Sweden	Swedestar project	unknown	GPs unknown	[96]
Switzerland	FIRE	113,335–293,000 (14–36%)	60–120 GPs	[97, 98]

^a^Percentage denominator (i.e., the country population was extracted from national official websites)

The number of participating GPs varied from 30 to 7200, depending on the country or project. This information was not retrievable for 17 projects. The investigators were often described as a group of healthcare professionals.

Most primary care data collection projects were implemented nationwide and not limited to a specific geographical location within a country. Nevertheless, several networks were based on location similarity, for instance the Melbourne East General Practice Network in Victoria [17], community health centers in Canada [18] or in the USA (for instance, greater Boston area [19] and New York [20, 21]). The EMRALD and DELPHI projects originated from Ontario, a Canadian province.

### Technological infrastructure of the IT systems used for data collection and data reuse

Technological infrastructures are key elements of routine data collection and reuse projects.

We distinguished these four items:

- EHR software with extraction tools
- Data warehouse, which import, classify and store data
- Functional integrated platforms, which gather tools to exploit data coming from the data warehouses
- Data linkage facilities, which give the possibility to cross data with other databases

They represent different levels of achievement of data collection and reuse projects.

#### EHR software tool as source of data collection

Most projects were set up in collaboration with a single EHR software company and a single IT system or software tool was used to initiate the routine primary care data collection project (Table 3). In the USA, the Veterans Health Administration was especially praised for developing an open source electronic medical records system: the Veterans Information Systems and Technology Architecture (VISTA). The choice of EHR vendor and the negotiations for the initial EHR software purchase were considered as the first major challenges in building the OCHIN database [22]. Moreover, the availability of many different software applications seems to hinder data collection. For instance, in Belgium, more than 17 different software systems are currently used by GPs and this hampered the development of primary care data collection projects, according to the authors [23]. Indeed, the use of different software applications increases the data collection complexity and adds interoperability issues. Nevertheless, some projects managed to collect data from different software systems with various database architectures. For instance, in the CPRD, primary care observational data came from three main EHR systems for GPs. However, historically, all data were retrieved from the Vision software [15]. For the CPCSSN, data were extracted from ten EHR systems [24]. The DELPHI program originally collected data only from the EHR software Healthscreen [25], but now contains data from various EHR software systems [24].

**Table 3:** Stakeholders

	Governmental services	Academic institutions	Software companies Private-sector companies
CPRD^a^	- Medicines and Healthcare products Regulatory Agency (MHRA) - Department of health via the Health and Social Care Information Centre (HSCIC) [15]	National Institute for Health Research (NIHR)	IMS Health© [180] Several EHR software systems [15]
THIN			In Practice System - Cegedim**©**, IMS Health**©** [16]
Q research		University of Nottingham and its division of Primary Care [42]	EMIS [42]
Veterans database^a^	Veterans Health Administration [152]		VISTA, developed by the Veterans Health Administration [152]
Geisinger© Health System project			Geisinger**©** Health system [30] IBM [162]
General Electric Centricity© electronic medical records research database			General Electric**©** [181]
OCHIN, Inc	US Agency for Healthcare Research and Quality Funding from the American Recovery and Reinvestment Act	OCHIN SafetyNetWest Practice-Based Research Network, non-profit partnership with academic researchers, governed by a board of directors	EpicCare [22]
Baylor Health Care System (BHCS) and Christiana Care Health System (CCHS)	Agency for Healthcare Research and Quality, US Department of Health and Human Services		One commercial EHR system [166]
University of California Davis Health System electronic medical record system		University of California [168]	University of California Davis Health System electronic medical record (Epic, Verona, WI)
CHCI			eClinicalWorks [170]
MGPO		Massachusetts General Hospital - Radiology Department associated with the Massachusetts General Physician Organization Primary Care Operations Improvement Group	
OpenDoor		Columbia University New York University School of Medicine Division of General Internal Medicine Mailman School of Public Health, Columbia University Primary Care Development Corporation	eClinicalWorks [173]
New York City Primary Care Information Project	Agency for Healthcare Research and Quality		eClinicalWorks [21]
IFH	Funding from New York state Health Resources and Services Administration		EpicCare ambulatory EHR [20, 176]
Department of Family medicine - Wisconsin	Wisconsin State Division of Public Health	Department of Family Medicine University of Wisconsin [178]	
CPCSSN^a^ and Delphi	Public Health Agency of Canada [24]	- Centre for Studies in Primary Care at Queen’s University [24] and several universities - College of family physicians [24]	Several EHR software systems [24]
EMRALD^a^	Ontario Ministry of Health and Long-Term Care, depending on the projects [74]	- Institute for Clinical Evaluative Sciences, a prescribed entity under Ontario’s Personal Health Information Protection Act [70] - University of Toronto and its Department of Family and Community Medicine - University of Ottawa	Several EHR software systems: Practice Solutions Software electronic medical record, Oscar [74]
IPCI^a^	No	Erasmus University Medical Center Rotterdam: Department of Medical Informatics, Unit of Pharmaco-epidemiology [29], Department of General Practice [29]	No
Nivel-PCD ^a^	Dutch Ministry of Health [41]	- Groningen University, Free University of Amsterdam, Utrecht University and more - National college of GPs - National association of GPs [41]	NIVEL owns the Information Communications Technology (ICT) department and has close links with vendors of ICT systems [41]
LINH	Netherlands Institute for Health Services Research (NIVEL) Dutch Ministry of Health [41]	- IQ healthcare - National association of GPs - Dutch College of GPs [41]	
*Julius* General Practitioners Network (*JGPN*) database^a^	No	- Julius Center for Health Sciences and Primary Care at Utrecht University Medical Center [39]. - GPs’ Networks at each Dutch University - Representatives of care groups (groups of GPs from the same area) have a seat in the scientific committee.	Information Communications Technology company (called Proigia) extracts primary care data from GP practices and send them to the database
RNH		Department of Family Medicine, subdivision of the Faculty of Health, Medicine and Life Sciences at Maastricht University. Maastricht University Medical Center. CAPHRI, school for public health and primary care [89]	
Transform project^a^	Depending on the country	Several universities worldwide (e.g., Crete, Antwerp) [79]	TransHIS Custodix, Belgium, for central data linkage
SIDIAP	Catalan Institute of Health [31]	- Jordi Gol Primary Care Research Institute - Girona Research Support Unit [31]	eCap, Catalan software company [31] IMS Health© [180]
ECRPC Madrid	Madrid Regional Health Authority		
BIFAP^a^	6 projects administered and financed by Agencia Española de Medicamentos y Productos Sanitarios (Spanish Agency of Medicines and Medical Devices), a public agency belonging to the Spanish Department of Health [95] - Institutional collaboration of the regional governments of the nine participant Autonomous Communities	- Department of General Practice - Learned societies of GPs’ support - One GP who is an active BIFAP collaborator appointed as member of the BIFAP Scientific Committee.	in-house IT and pharmaco-epidemiologist teams No private-sector company
GRAPHC		- Australian National University - Australian Primary Health Care Research Institute [182]	
Canning Division of General Practice		Australian General Practice Network	
MEGPN	Independent accredited not-for-profit organization [27]		PCS Clinical Audit Tool© Practice Health Atlas©
Intego^a^	Flemish government, Department of Health Care [54]	- Department of General Practice of the KU Leuven [54] - Other universities. - GPs’ participation in the ethics and scientific committee	- Datasoft, Medidoc - Careconnect EHR - No private-sector company involved in data analysis
NIHDI	Public social security institution [23]		Extraction module on EHR [23]
Health Search Database / CSD©			Cegedim**©** [77]
Swedestar project		- Department of Medical and Health Sciences, University of Linköping, Unit of Research and Development in Primary Care, Jönköping - Lindsdals Primary Health Centre [96]	Swedestar software [96]
FIRE		- Institute of General Practice of Zurich University [97] - “SGAM informatics”, a study group of the Swiss Association of GPs [26]	Grhanite extracting tool
LPD network - Cegedim			Doc’ware software - Cegedim**©** [8]

^a^the contact person gave information on the project governance following our enquiry by email

Software development companies, which produce the EHR software, were usually requested to implement the data extraction tools. In Belgium, EHR software developers were asked to develop an extraction module to support NIHDI uploading procedure [23]. In Switzerland [26], the companies that provided EHR software for the FIRE project were asked to update their products with an exporting tool (called Ghranite) to enable the automatic downloading of core FIRE variables from individual EHRs to a central server [27]. The OCHIN project also stressed the importance of finding a software vendor who was willing to provide system modifications and enhancements.

Finally, offering simplified data extraction tools to minimize the additional workload is a major factor. Otherwise, the applicability and utility of EHR data for large-scale research purposes remains limited as pointed out by most of the project websites.

#### Implementation of comprehensive data reuse platforms

The technical architecture of the IT system for data reuse was poorly detailed in all articles. However, we could extract some trends concerning the delivery of data mining solutions.

Considering the aim of routine data collection projects is to analyze data, this part is essential to expand data exploitation and bring data reuse to a higher level of use.

##### A centralized official data warehouse

A data warehouse has several roles: it imports, classifies and stores data coming from the EHR software tool.

In most cases, the data warehouse was linked to an official administration (university, national administration warehouse) that brought the added value of official recognition. For instance, in the Q research project, the EMIS software transmitted in a secure way all aggregated data to the University of Nottingham that was and still is the only access point to the full database. In the USA, the Veterans Administration built the Corporate Data Warehouse and four Regional Data Warehouses to provide a standard architecture that centralizes all clinical data [28]. In the Netherlands, most GP databases, including IPCI, are connected to one of the eight national Medical University Centers [29].

##### A functional integrated platform: A turnkey service

We define as functional integrated platforms, services which gather tools to exploit data coming from the data warehouse.

According to the official websites of the major primary care data collection projects identified in this analysis (i.e., CPRD, THIN, Veterans Administration database and CPCSSN), they facilitate data exploitation by providing networks that extract datasets from the data warehouse for researchers and by offering a range of services and products in the areas of medical research and public health care. For instance, in the USA, data retrieved from healthcare facilities have been made available for research purposes in a data warehouse (corporate data warehouse). On top of this warehouse, standard tools were developed for robust access, reporting and data analysis at the enterprise level. The Veterans Affairs Informatics and Computing Infrastructure (VINCI) is an initiative to improve the researchers’ access to Veterans Affairs data and to facilitate their analysis, while ensuring data privacy and security [28].

Besides these global extraction systems, few modules have been developed with specific functionalities related to research. For instance, the CPRD software system is integrated with EHR systems to enable randomization at point of care in a real world setting [15].

##### Integrating data linkage functionalities

Data linkage facilities give the possibility to cross data with other databases. To expand the data processing potential, linkage with other databases has been included in the integrated IT systems used for these projects. CPRD services were progressively developed to increase the primary care data coverage and the number of linked datasets [15, 16]. For instance, CPRD GOLD, the primary care database of CPRD, has access to the following linked datasets: Death Registration data from the Office for National Statistics, Cancer Registration data from Public Health England, Hospital Episodes Statistics data. The Geisinger**©** Health System warehouse receives feeds from multiple-source systems, including data from EHRs and also data on financial decision support, claims and patient satisfaction [30]. SIDIAP allows linking to other Catalonia databases [31], such as CMBD-AH (dates, diagnoses and procedures linked to admissions in each Catalonia hospitals) and the death registration database (date and causes of death of all Catalonia residents). Globally, data linkage with other databases has been developed by many IT systems to expand the potential of their network. Moreover, as highlighted by Phillips and colleagues, “Integrated big data systems could also collect information directly from patients about their health behaviors, community resource utilization, social networks, and other social determinants of health” [32]. Similarly, the University of Wisconsin Electronic Health Record-Public Health Information Exchange (UW eHealth-PHINEX) can be used to study “health and disease within the patient’s biologic, psychosocioeconomic, environmental, and community context” [33]. In this database, EHR data can be linked to geographic, environmental, socioeconomic, and demographic statistical data to obtain comprehensive information for the investigation of infectious, acute, chronic, injury-related, occupational and environmental health outcomes and risk factors. Then, it allows using multivariate analysis and data mining tools to identify variables for predicting disease and health quality at the census block level.

### General practitioners’ involvement in the development of data collection networks

#### GPs as care pathway coordinators

Gate-keeping is the identification of patients with one primary care provider who is most responsible for their care. Gate-keeping by GPs has been recently adopted in most European countries and in many countries worldwide. The inherent central place accorded to GPs enhances their key role in “leading primary care and community big data efforts” (172). The GPs’ key role in the development of data collection networks was particularly highlighted in the Canadian primary care data collection projects. In Ontario, following primary care reforms, patient rostering (i.e., the connection of one patient to one physician or physician team) has been introduced [34] and more than 80% of the population is rostered to a primary care physician [35]. Conversely, other Canadian provinces do not currently have similar broadly based primary care patients’ enrollment systems [36]. Analysis of the retained articles indicated that primary care data collection networks (DELPHI, EMRALD) are most developed in Ontario.

#### Role of the type of primary care organization

Analysis of the selected articles indicated that primary healthcare organizations differ among countries, from GP’s office to outpatient clinics. However, the type of primary care organization (for instance, small GP practices in the UK and outpatient clinics in the USA) did not seem to influence the development of successful primary care data collection projects.

#### GPs’ contribution and incentives

GPs voluntarily agree to supply data to the data collection project. Sometimes, their contribution required high quality and completeness standards [16, 37], coding accuracy [38], or seniority in software use [34, 37]. Moreover, incentives may be offered to improve GPs’ contribution and compliance. For instance, data providers receive a percentage of the profits generated by research carried out using THIN [16]. The Julius network also compensates financially the time spent in supplying data [39]. In addition, GP practices involved in the CPRD project can generate research revenues through their involvement in studies that require validation, sample collection, or patient questionnaires [15]. Similarly, the THIN project offers opportunities for extra payments to GPs when researchers require supplementary data about selected patients [16]. Conversely, Q research is a not-for-profit network and does not have funding to compensate GPs for their contribution [40].

Software training sessions also have been proposed to GPs involved in data collection for the THIN or Julius network [16, 39, 41]. Moreover, regular feedback and reports on data recording might be given to the participating GPs [16, 39, 41]. Indeed, GPs are increasingly interested in using their EHR data to better understand and manage their patient populations. GPs are also encouraged to start their own research project [42].

### Stakeholders of routine primary care data collection projects

The contact information of 36 primary care data collection projects was used to enquire about the project managers by email. Based on their responses (*n* = 10) and the information found in the articles or websites, we were allowed identifying three major project governance actors:

- governmental services (Veterans Health Administration in the USA, National Health Service in the UK)
- academic institutions (universities and department of general practice) and GP representatives (Nottingham University, Department of General Practice of Leuven University, Dutch College of General Practitioners)
- private-sector companies specialized in software development (Cegedim^©^) or in data extraction and analysis (IMS Health^©^)

The collected information highlighted that only few projects were exclusively managed by software companies. IMS Health^©^, which bought Cegedim^©^ Customer Relationship Management Software and Strategic Data in 2015, is the leader in several countries. We noted that the partnership between CPRD and IMS Health^©^ was not mentioned in the CPRD website or in the reply following our enquiry by email, but was quoted in the IMS Health^©^ website.

On the other hand, most projects were supported by academic or governmental institutions in association with software companies (particularly for the extraction process). Generally, universities were involved via their general practice or medical informatics departments. Governmental services also were involved, but their role (funding, ethical validation) was often not specified. Concerning governmental services, a unique official initiative is the Health Information Technology for Economic and Clinical Health (HITECH) Act in the USA that defines the direct involvement of the state in the development of data reuse. More precisely, it supports the meaningful use of certified EHR technology, “connected in a manner that provides for the electronic exchange of health information to improve the quality of care; and provides to the Secretary of Health & Human Services (HHS) information on quality of care and other measures” [43].

## Discussion

This review of the literature allowed the identification of determinants that favor the development of durable routine data collection projects.

### Methodology

#### Study strengths

This is the first review that lists the databases derived from primary care electronic health records and their organizational features.

Moreover, we followed the PRISMA criteria [12], an evidence-based minimum set of items for reporting in systematic reviews. Then, quality was controlled using the AMSTAR checklist [13].

#### Study limitations

##### Methodological issues

The aim of this study was to identify the determinants within the healthcare organization that influence routine data collection projects in primary care. We could identify 36 projects by screening the PubMed database. We only used this database, but added information from the gray literature.

##### Missing or partial data

We took for granted that the PubMed occurrences were correlated with the international outreach of primary care databases. This hypothesis was validated by the fact that our query identified major routine primary care data collection projects.

Nevertheless, there is actually a discrepancy between the effective number of publications of each project and the number of publications retrieved in our paper. There are several reasons to explain the differences.

- Firstly, our query search filters by the publication year. CPRD database was created in the 1980s, so numerous papers are not taken into account by our query.
- Secondly, publications listed on the websites are not indexed into PubMed.
- Thirdly, due to the lack of MeSH term to index primary care databases, our query search was not able to retrieve all papers published. Indeed, if we analyze the PubMed keywords of articles that were not returned by our query but which were recorded on database websites, you can note that they are not referenced as primary care data collection projects, but as the main topic of the article. For instance, the paper “Prevalence, incidence, indication, and choice of antidepressants in patients with and without chronic kidney disease: a matched cohort study in UK Clinical Practice Research Datalink.” is indexed with the keywords “antidepressants; chronic kidney disease; depression; incidence; prevalence”.

Finally, our initial purpose was to find papers describing the characteristics of the data mining projects and not all the results of the exploitations of such database. Including all the publications was not achievable because all websites do not reference their publications and it was not answering our initial methodology. Concerning the project description, the number of patients was difficult to extract from the articles because several studies were based on subsets of the available databases. We finally chose to take into account, when possible, the figures indicated in the website because it described the whole project. It would have been interesting to compare projects according the patient-year data, but this figure was rarely available.

Another issue was the number of GPs per project. Indeed, the method of representation of healthcare professionals varied in the different countries (e.g., number of GPs, number of healthcare professionals, number of community practices). We observed that the number of patients was not proportional to that of GPs, suggesting that the GPs’ involvement varied in function of the project nature or the primary care organization of the specific country. Moreover, the number of participating GPs could be different according to the chosen data mart [23].

There was no systematic correlation found between the representativeness of population in the database, the number of GPs and the number of publications per database. We thought that the larger the database is, the more publications are edited. But, the amount of data can also be an hindrance and without comprehensive data formatting, data mining turns out to be more complex. This result could invite us to begin with the creation of a local routine data collection project before expanding it to a larger scale in our country.

We reviewed each article and website using a predefined form (Fig. 2). Nevertheless, some data were missing especially about stakeholders.

Finally, this study focused on successful data collection projects and therefore we could not identify factors that could limit GPs’ participation, such as privacy issues, lack of training and information.

### Summary of evidence

#### Geographical distribution of routine primary data collection projects

This study highlights the leadership of English-speaking countries: UK, USA and Ontario, in Canada, are clearly ahead of European countries.

Moreover, geographical proximity was sometimes important for setting up and implementing a project, for instance the EMRALD project. Similarly, in France, hospitals from the same area often pool together their information to aggregate a larger database [3, 4], and routine primary data collection projects and initiatives are primarily local (only one national project) [3, 9]. Nevertheless, geographical proximity is then pushed into the background by the virtual nature of electronic information. Thus, the network effect seems to be a facilitating, but not a determining factor.

#### The major role of EHR software in data extraction/collection

When it comes to the implementation of a routine primary data collection network, software companies play a key role directly via their software system and the development of data extracting tools. Most of the reviewed projects included at least one software company, suggesting that they are part of the foundations of such projects.

Moreover, most projects were linked to a single software application, thus limiting interoperability issues and technically facilitating data analysis. This suggests that the number of software systems hinders the development of a primary care data research networks. Indeed, interoperability issues hinders the development of a unique database. Data sources come from EHR and the presentation of data can be heterogeneous considering each software application has its own logic and data schemas. Data are not identically formatted and thus are not always comparable. In France, more than 12 software applications are used [44] and this could explain the difficulty to create a nation-wide primary care data collection network.

#### A comprehensive IT system dedicated to data exploitation

Our study also highlights that data must be processed and integrated before being released to final users, generally academic researchers. Projects often offer support to researchers for data extraction and analysis. The provision of data sources (EHR data) requires formatting and processing before being included in the data warehouse. Then, a second step consists of the extraction of datasets from the data warehouse. Enabling users to shuttle data sets of any type seems difficult considering the large amount and complexity of data. Indeed, the big data area requires a deep knowledge of the content and form of the data handled.

This implies the development of a comprehensive IT platform to provide services and tools to end users. In France, we combined secondary care data with administrative information from the National Health Insurance Cross-Scheme Information System. This led to the development of a global IT system that includes a data management platform where data are available to researchers via dedicated tools [2].

#### GPs: Care pathway coordinators who are essential for data collection

##### Factors enabling GPs involvement

The organization of primary care varies depending on the countries (from isolated GPs to outpatient clinics), but this does not seem to affect the development of primary care data collection projects. Particularly, routine primary data collection projects are not more numerous in countries with big primary care facilities. Nevertheless, a local network effect can occur and facilitate the spreading of such initiatives.

In addition, several strategies are often put in place to increase and optimize GPs contribution. GPs participate voluntarily, but their participation can be promoted by the various advantages they can have in return: financial benefits, training sessions, regular feedback, and participation in research programs. These benefits could be proposed also to French GPs to create a French primary care data collection project.

Training sessions for GPs included data coding training because most of the data are coded using International Classification of Primary Care version 2 (ICPC-2). We think that training GPs in data coding (basic and continuous training) should have a positive influence on their participation.

Moreover, most projects/networks give regular feedback reports on data recording to GPs [16, 39, 41]. In France, this feedback could represent a complementary method to assess the data required for calculating the GPs’ remuneration based on public health objectives. Moreover, they could improve GPs’ knowledge of their activity. Indeed, GPs are increasingly interested in data reuse at an individual level as described in the next section.

##### GPs role in the global network organization

First, GPs are essential data providers. As care pathway coordinators [6], they represent an access point to the medical history of most of the population. GPs are by definition key players in the data collection process of primary care routine data collection networks. In France, GPs are also care pathway coordinators and therefore, this can help the development of primary care data collection projects.

Then, their role is proactive in the extraction project so as to improve data quality, that is to say reliable and reproducible data. Not only do they provide data, but they also ensure data quality. They can benefit from software training sessions and they often provide formatted or coded data to data warehouses.

Besides, GPs are also interested in data reuse at an individual level. Thanks to training, they learn to code their information and ease their abilities to extract information about their patients. GPs involved in the CPRD extraction process can receive regular practice-level quality improvement feedback, helping them improve clinical outcomes for their patients [15]. For instance, feedback on antibiotics use can be interesting so as to analyze medical practice. In the context of pay for performance, a better understanding of their patients can allow them to benefit from financial incentives.

Moreover, they can be involved at other levels of the network. For instance, department of family medicine are often members of the routine data collection networks as shown by our analysis of stakeholders in section “Stakeholders of routine primary care data collection projects”. Thus, they are directly implicated in data reuse process via the selection of the data extracted and the knowledge necessary around these data. Indeed, data mining on a large scale requires a deep knowledge of the data analyzed, via the development of quality control tools. It also requires expertise to generate relevant research questions.

Lastly, due to the privacy-sensitive nature of the information, GPs represent one of the guarantees of the integrity of routine data collection projects. This intrinsic feature facilitates the adhesion of patients and other GPs, to achieve and maintain their partnership.

#### Governance differences

Governance relates to "the processes of interaction and decision-making among the actors involved in a collective problem that lead to the creation, reinforcement, or reproduction of social norms and institutions." [45]. We could identify three major actors that support primary care data collection projects: private-sector companies, governmental services and academic institutions.

##### Private-sector companies: From extraction tools to privately funded collection projects

In the absence of publicly funded software tools, such as the American VISTA software, the involvement of private-sector software companies seems to be unavoidable. We observed various levels of implications.

First, EHR software companies are involved in the development of extraction tools. Indeed, data transfer depends on the database scheme that is only known by software companies. After extraction, data are normally analyzed by academic partners, often including GPs. Indeed, very few projects were completely (data extraction and analysis) managed by private-sector companies, without the help of other official parties. For instance, IMS Health© extracts, mines and commercializes health information. In France, Cegedim© retrieves EHR data that have been coded by GPs. In such cases, the GPs’ role is limited to supplying data and coding some information.

##### Governmental services and academic institutions

The review of the main primary care data collection projects highlighted that frequently governmental services establish partnerships with academic institutions and that GPs are often part of an academic partnership. The specific role (funding, ethical assessment or data warehousing) of each party was difficult to determine.

The USA recently chose to legislate to promote data reuse; however, in our study we could not determine the effect of such legislation on the development of routine primary care data collection projects. Information was rarely available on USA data warehouses and we did not receive any answer to our enquiry by email.

Universities via their Department of General Practice are stakeholders in many projects. This involvement represents GPs’ interests, and can also promote GPs’ participation and be source of research project proposals, strengthening the GPs’ involvement from data extraction up to data mining. Moreover, the partnership with university departments connects GPs with official institutions and with the academic community.

In this review, we identified elements that influence the development and durability of large data collection projects. It will be interesting to extend and deepen our knowledge of these databases: their purpose, content, form (structured or free-text data), quality. Indeed, in the age of Big Data, new methods of data analysis are appearing. Artificial intelligence and its deep learning methods can not only provide clinical decision support systems [46, 47] but also abilities to analyze free-text information from a large amount of data through new natural language processing algorithms.

Furthermore, the management of privacy, which is a major deterrent for GPs, is still to be addressed and should also be studied.

## Conclusion

We performed a systematic review with the aim of determining the factors that allow the creation and expansion of routine data collection projects in primary care.

Technological infrastructure influence the outreach of data collection projects, particularly EHR software tools for data extraction. Beyond this first step, the most successful projects also developed comprehensive IT platforms for research purposes.

As GPs are often care pathway coordinators in most countries, primary care data are particularly important for improving healthcare management. Therefore, GPs’ investment in these projects is often promoted with financial benefits, training sessions, feedback reports and involvement in research studies.

Finally, we emphasize the concomitant involvement of three main actors in supporting these initiatives: governmental services, academic institutions and software companies. Their partnership seems to be the most effective way to fund long-term and wide range data collection projects.

Several issues still need to be addressed, such as the nature of the data analyzed (coded or free-text data) or the management of privacy, which is a major deterrent for GPs. As the collected data are very sensitive, careful monitoring should be put in place due to the privacy issues at stake. Patients’ de-identification should also be studied. Moreover, the project governance and its managers should be extremely transparent to improve GPs’ adhesion to routine primary care data collection projects.

The table does not include isolated projects that did not refer to reusable databases, and the TRANSFoRm project, which is a multinational project within Europe based on national databases, such as Nivel in the Netherlands.

## Abbreviations

BHCS: Baylor Health Care System
CCHS: Christiana Care Health System
CHCI: Community Health Center, Inc.
CPCSSN: Canadian Primary Care Sentinel Surveillance Network
CPRD: Clinical Practice Research Datalink
DELPHI: Deliver Primary Healthcare Information
EHR: Electronic Health Record
EMRALD: Electronic Medical Record Administrative Data Linked Database
FIRE: Family Medicine ICPC-Research using Electronic Medical Records
GP: General Practitioner
GRAPHC: Geographical and Resource Analysis in Primary Health Care
IFH: Institute for Family Health
IPCI: Integrated Primary Care Information project
JPCRN: Julius Primary Care Research Network
MEGPN: Melbourne East General Practice Network in Victoria project
MGPO: Massachusetts General Physicians Organization
OCHIN: Inc. Oregon Community Health Information Network
PHINEX: University of Wisconsin Electronic Health Record - Public Health Information Exchange
THIN: The Health Improvement Network
UK: United Kingdom
USA: United States of America
